# Disease Surveillance during the Reintroduction of the Iberian Lynx (*Lynx pardinus*) in Southwestern Spain

**DOI:** 10.3390/ani11020547

**Published:** 2021-02-19

**Authors:** Fernando Nájera, Rebeca Grande-Gómez, Jorge Peña, Anastasio Vázquez, María Jesús Palacios, Carmen Rueda, Ana Isabel Corona-Bravo, Irene Zorrilla, Luis Revuelta, María Gil-Molino, José Jiménez

**Affiliations:** 1 Department of Animal Physiology, Faculty of Veterinary Medicine, Complutense University of Madrid, 28040 Madrid, Spain; lrevuelt@vet.ucm.es; 2 Asistencia Técnica de la Dirección General del Medio Natural y Desarrollo Sostenible de la Junta de Comunidades de Castilla-La Mancha, Plaza del Cardenal Siliceo s/n, 45071 Toledo, Spain; 3 GPEX-Dirección General de Medio Ambiente, Junta de Extremadura, Avda. Luis Ramallo s/n, 06800 Mérida, Spain; rgrandegomez@gmail.com (R.G.-G.); jorgepmartinez@gmail.com (J.P.); tasiopeseto@gmail.com (A.V.); 4 Organismo Autónomo Parques Nacionales, Zarza de Granadilla, 10710 Cáceres, Spain; 5 Dirección General de Medio Ambiente de la Junta de Extremadura, Avda. Luis Ramallo s/n, 06800 Mérida, Spain; mariajesus.palacios@juntaex.es; 6 Fundación CBD-Hábitat, C/Gustavo Fernández Balbuena 2, Entreplanta, Oficina A, 28002 Madrid, Spain; carmen.rueda91@gmail.com; 7 Centro de Análisis y Diagnóstico de la Fauna Silvestre, Agencia de Medio Ambiente y Agua de Andalucía, Consejería de Agricultura, Ganadería, Pesca y Desarrollo Sostenible, Junta de Andalucía, Avenida Lope de Vega 9, 29010 Málaga, Spain; aisabel.corona@juntadeandalucia.es (A.I.C.-B.); irene.zorrilla.delgado@juntadeandalucia.es (I.Z.); 8 Servicio de Recepción y Diagnostico de Muestras Biológicas, Hospital Clínico Veterinario, Universidad de Extremadura, Avda. Universidad s/n, 10003 Cáceres, Spain; magilmo84@gmail.com; 9 Instituto de Investigación en Recursos Cinegéticos-(CSIC-UCLM-JCCM), 13071 Ciudad Real, Spain; Jose.Jimenez@uclm.es

**Keywords:** Aujeszky’s disease, canine distemper virus, disease surveillance, feline leukemia virus, *Lynx pardinus*, reintroduction

## Abstract

**Simple Summary:**

The restoration of Iberian lynx (*Lynx pardinus*) populations in Extremadura (Southwestern Spain) have been carried out since 2014. To evaluate the effect that infectious diseases may have on their reintroduction, we performed a molecular and sero-epidemiological survey in reintroduced and wild-born lynxes and sympatric carnivores. From 2015 to 2019, 69 Iberian lynxes were screened against 10 viral, bacterial and piroplasmid agents. In parallel, 195 sympatric carnivores were tested against current or past infections to six common canine/feline viruses. In the Iberian lynx, low contact rates of active infection were obtained for feline leukemia provirus (FeLV: 1.5%; 1/67), feline parvovirus (FPV: 1.5%; 1/67) and *Cytauxzoon* sp. (6.7%; 1/15). We confirmed the emergence of Aujeszky’s disease (suid herpesvirus-1) in this population (SuHV-1: 11.8%; 2/17). We detected the circulation of FeLV, parvovirus, canine distemper virus (CDV), feline calicivirus (FCV) and feline immunodeficiency virus within the sympatric carnivore community and FCV, FPV, CDV and feline coronavirus in lynxes. Due to the low contact rate of infectious agents in such a small, endangered population, we recommend continuing a disease surveillance program to determine the prognostic factors of survival, understand the role that disease may play during the reintroduction and anticipate disease outbreaks that may pose a risk for the entire reintroduced population.

**Abstract:**

The restoration of Iberian lynx (*Lynx pardinus*) populations in Extremadura (Southwestern Spain) have been carried out since 2014. One of the measures to ensure the success of this program is to examine the effects that diseases may have on reintroduction. Since diseases may be greatly located at certain sites because of the specific ecological requirements of the pathogens and/or vectors, reintroduced individuals may present a risk of infection once released. To determine which pathogens the reintroduced individuals may encounter, we performed a molecular and sero-epidemiological survey in reintroduced and wild-born lynxes. From 2015 to 2019, 69 Iberian lynxes (40 reintroduced and 29 wild-born) were sampled and screened against 10 viral, bacterial and piroplasmid agents. In parallel, 195 sympatric carnivores from the families *Canidae*, *Felidae*, *Viverridae*, *Herpestidae* and *Mustelidae* were tested against current or past infections to six common canine/feline viruses. In the Iberian lynx, low contact rates of active infection were obtained for the feline leukemia provirus (FeLV: 1.5%; 1/67), feline parvovirus (FPV: 1.5%; 1/67) and *Cytauxzoon* sp. (6.7%; 1/15). We also confirmed the emergence of Aujeszky’s disease (suid herpesvirus-1) in this population (SuHV-1: 11.8%; 2/17). Evidence of previous exposure was detected for canine distemper virus (CDV: 5.8%; 3/52), feline coronavirus (1.9%; 1/52), FPV (7.7%; 1/13) and feline calicivirus (FCV: 5.3%; 1/19). From 25 recovered lynx carcasses, we could confirm infectious etiology involvement in the death of four individuals (SuHV-1 in two individuals, coinfection of *Cytauxzoon* spp. and *Aeromonas veronii* in one lynx and a *Streptococcus canis* myositis in another lynx). We confirmed the circulation of CDV, FPV, FeLV, FCV and the feline immunodeficiency virus within the sympatric carnivore community. Due to the low contact rate of infectious agents in such a small, endangered population, we recommend continuing a disease surveillance program to determine the prognostic factors of survival, understand the role that disease may play during the reintroduction and anticipate disease outbreaks that may pose a risk for the entire reintroduced population.

## 1. Introduction

Carnivores play important and unique roles in the natural functioning of ecosystems [1]. Despite their roles, more than half of the world’s largest carnivores are threatened with extinction [2]. To reverse this situation, reintroduction has become an increasingly popular conservation technique [3,4,5]. The endangered Iberian lynx (*Lynx pardinus*) is a trophic-specialist felid considered an apex predator [6] that has been absent from Extremadura (Southwestern Spain) since the late twentieth century [7,8]. 

Since 2014, efforts to restore Iberian lynx populations have been carried out within the framework of the project LIFE+10NAT/ES/570, which aims to recover the historical range of the species in Spain and Portugal. In Extremadura, since the initial release of 45 individuals between March 2014 and April 2019, the population increased to ca. 95 individuals by December 2019.

During the initial phases of restoration, the small number of reintroduced individuals may be largely threatened by the effects of disease. A valuable program for health risk assessment for a carnivore restoration has two parts: evaluating the health of animals to be released and evaluating the health risks at the release site [9]. As health risk assessment is dynamic, emerging health risks should be monitored in the reintroduced population and sympatric species to provide an insight into future mortality events of the released individuals [9]. Regarding infectious diseases, mammals from clades that are closely related to domesticated animals are at the greatest risk of parasite-mediated declines, most likely due to the cross-species transmission of generalist viruses and bacteria [10]. Reintroduced species may be particularly vulnerable to parasite invasion and the adverse effects thereof [11]. Reintroduced host populations may have no/low herd immunity towards native, circulating parasites, resulting in more explosive and severe infections than under endemic conditions [12].

In the case of the Iberian lynx, previous studies in the two remnant wild populations from Andalusia showed low contact rates with viral pathogens, which might make the lynx vulnerable to outbreaks of certain diseases due to a lack of acquired immunity [13,14]. Moreover, a mortality survey carried out from July 2006 to December 2011 showed that the most common cause of death registered in those population nuclei was infectious diseases, which accounted for as much as 38.5% of all recorded mortalities [15]. Among all other pathogens, the Feline Leukemia Virus (FeLV) has shown to be critical in the Iberian lynx population since the emergence of an outbreak in 2007 in Doñana, one of the last two strongholds of the species at that time [16]. That aggressive outbreak killed two-thirds of the infected lynxes, probably due to increased host susceptibility to pathogens [17,18], since the FeLV sequences isolated from that outbreak revealed their relationship with naturally occurring FELV-A infections in domestic cats [18]. Other viral infections of concern in this species include parvovirus, canine distemper virus (CDV) and Aujeszky’s disease (SuHV-1), since they could be fatal in the Iberian lynx [15,19,20].

Here, we provide information pertaining to the infectious diseases screening of released and wild-born Iberian lynxes and the infectious disease burden in the sympatric carnivore community during the first five years of the Iberian lynx reintroduction program of the Autonomous Region of Extremadura (SW Spain). In general, we focused on multi-host pathogens that may cause morbidity and/or mortality in lynxes. We also explored the role of the sympatric carnivore community as a disease reservoir throughout the reintroduction landscape.

## 2. Materials and Methods

**Figure 1:**
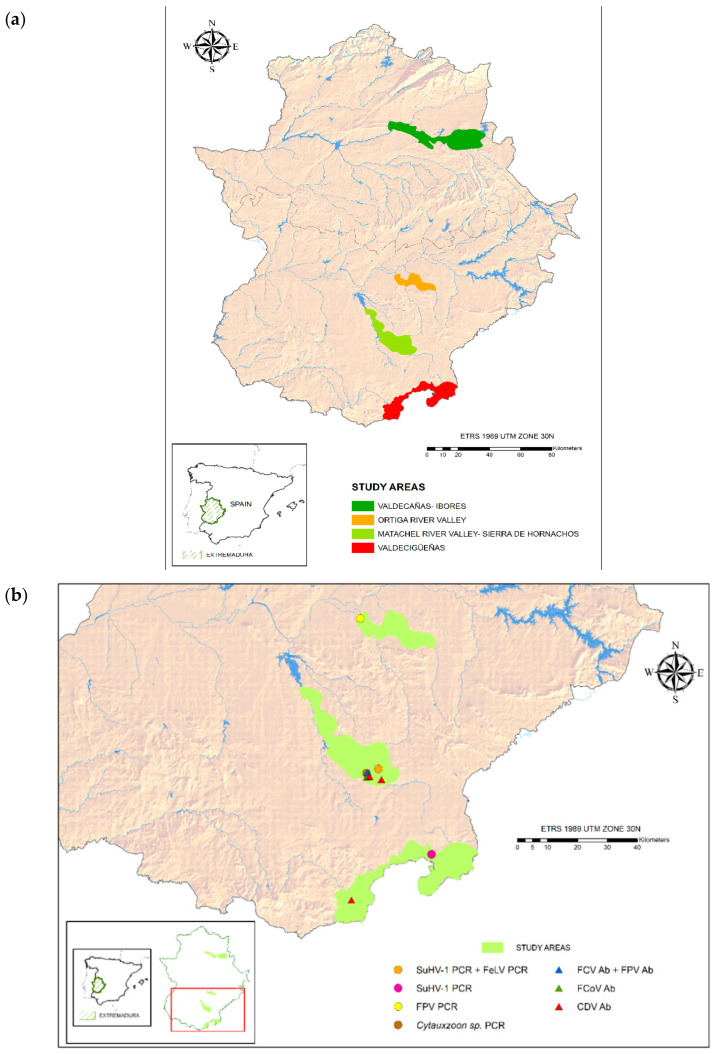
(**a**) Study areas within the Extremadura Iberian lynx reintroduction program. (**b**) Location of pathogen-positive lynxes by PCR and serology (Ab = antibodies). SuHV-1: suid herpesvirus-1; FPV: feline parvovirus; FCV: feline calicivirus; FCoV: feline coronavirus; CDV: canine distemper virus.

The study was carried out in Extremadura (SW Spain), consisting of four sites: (1) Matachel River Valley-Sierra de Hornachos (MRV), (2) Ortiga River Valley (ORV), (3) Valdecigueñas (V) and (4) Valdecañas-Ibores (VI) (Figure 1). Two reintroduction sites (MRV and ORV) were selected according to four factors: main prey (European wild rabbit) availability, suitable habitat availability, minimum continuous surface area of ca. 10,000 ha, and meta-population integration possibilities, which allow to obtain a viable lynx population in the long term (for further information, see http://www.iberlince.eu/images/docs/3_InformesLIFE/ProtocoloSeleccionAreas_M.Iberlince.pdf accessed on 17 October 2019). The other two sites (V and VI) were selected after the establishment of dispersing lynxes in these areas. Those sites consist mainly of private hunting estates and protected areas, with villages in and around those sites. The altitude ranges between 346–667 m above sea level. The landscape is a mixture of cultivated lands, open oak woodlands (“dehesa”) and scrubs. Vegetation is dominated by holm oak *Quercus ilex* and olive *Olea europea* trees, with a shrub layer of Mediterranean maqui scrubland (e.g., *Erica* spp., *Cistus* spp. and *Rosmarinus* spp.) and dense scrub (*Pistacia lentiscus*, *Quercus coccifera* and *Flueggea tinctoria*) but, also, open pasture areas. The main land uses include extensive farming (cereal crops and vineyards), livestock farming and estates managed for large and small game hunting.

From March 2015 to December 2019, we sampled 69 free-ranging lynxes (40 captive-born and 29 wild-born lynxes) either captured during the trapping season (September–December each year, *n* = 44) or found dead (*n* = 25) during daily field operations. Samples from 29 females and 40 males were included in this study. Within the age category, 31 samples corresponded to adults, 18 to subadults and 20 to juvenile lynxes.

Captive-born released lynxes came from the Ex-situ Conservation Program [21]. Prior to release, lynxes tested negative against CDV, FeLV, feline immunodeficiency virus (FIV), feline calicivirus (FCV), feline coronavirus (FCoV), feline parvovirus (FPV) and feline herspesvirus-1 (FHV-1) by PCR. They also received a vaccination booster against FCV, FHV-1, FPV, FeLV and *Chlamydophila felis* (Fevaxyn Pentofel, Zoetis, Belgium and FeLV PureVAX, Merial, France). All the released animals were fitted with telemetry collars and were monitored. Captive-born released lynxes were captured for a health evaluation and/or radio collar change at least one year after their reintroduction. Wild-born lynxes were detected via camera-trapping or direct sightings. Lynxes were captured using a single-door cage trap baited with rabbit. All individuals were anesthetized using a mixture of dexmedetomidine-midazolam-ketamine and supplemented with isoflurane inhalatory anesthesia if needed [22]. Anesthetized Iberian lynxes underwent a complete routine health evaluation. Blood (10–14 mL) was obtained by femoral, cephalic or jugular venipuncture and collected in EDTA-coated tubes, lithium heparin-coated tubes and serum separator tubes (Aquisel, Selecta Group, Barcelona, Spain). Blood collected in serum separator tubes was allowed to clot and was then centrifuged at 50 *g* for 15 min. The serum was removed and frozen at 20 °C until analysis. Swab samples using specific media for viruses were taken from the oro-pharyngeal cavity and rectum and preserved frozen at 20 °C until analysis. Lynxes (*n* = 60) were tagged with VHF (Wagener, Köln, Germany) or VHF-GPS-GSM (Sirtrack G3C, Sirtrack Wildlife Tracking Solutions, Hawkes Bay, New Zealand, Tellus Ultra = light/Televilt/TVP positioning Followit AB, Lindesberg, Sweden and Microsensory, Fernán Núñez, Spain) collars. Finally, after handling, all individuals were safely released at the capture site.

Dead lynxes were found by (1) the mortality signal of the collars, (2) direct citizen sightings (e.g., roadkill) or (3) following an investigation (e.g., poached individuals). Lynx carcasses were then transported to the Wildlife Rescue Center of Los Hornos (Caceres, Spain) and followed a standard necropsy procedure usually within 6–24 h after their discovery. Samples from the main tissues (spleen, mesenteric ganglia, bone marrow, liver, kidney, large intestine and cerebrum) and blood from cardiac puncture were obtained and frozen at 80 °C until analysis. Additional testing such as bacterial culture were also performed if bacterial colonization or septicemia was suspected.

A total of 195 sympatric carnivores were sampled between January 2014 and June 2019. Samples came from live-captured (*n* = 105) or road-killed/hunted (*n* = 64) free-ranging individuals. Species sampled in this study included feral cats (*Felis catus*, *n* = 75), rural dogs (*Canis lupus familiaris*, *n* = 26, sampled with owners’ consent), Egyptian mongoose (*Herpestes ichneumon*, *n* = 27), red fox (*Vulpes vulpes*, *n* = 36), stone marten (*Martes foina*, *n* = 17), common genet (*Genetta genetta*, *n* = 13) and Eurasian badger (*Meles meles*, *n* = 1).

Free-ranging carnivores were captured using commercial cage traps (Tomahawk models 108 and 207, Tomahawk Live Trap Co., Tomahawk, Hazelhurst, WI, USA and Safeguard model 52824, Safeguard Products/Valco Companies, Inc., New Holland, PA, USA) baited with rabbit, hare, partridge or poultry. Once captured, they were anesthetized using different drug combinations depending on the species (e.g., medetomidine-ketamine or medetomidine-tiletamine-zolazepam). Blood (1–3 mL) was obtained from the cephalic or jugular veins and collected in EDTA-coated tubes and/or serum separator tubes. Blood and serum handling followed the same protocols as described above.

Dead-found carnivores were subjected to a field postmortem examination. Blood from cardiac puncture was obtained and frozen at 80 °C until analysis.

We performed a molecular survey for CDV on all species; FeLV on lynxes and domestic/feral cats and FHV-1, FCV, FPV, FIV, FCoV and SuHV-1 on *Cytauxzoon* sp. and *Lepstospira* spp. in lynxes. We also determined the exposure of lynxes and feral cats to FCV, FHV-1 and FIV and the exposure in all species to canine distemper virus and parvovirus (feline or canine). Vaccinated dogs and lynxes were not included in the serology analysis with respect to the agent(s) against which they were vaccinated. Due to limited serum quantities, not all tests were performed on all the individuals.

The summary of the methodologies used to detect evidence of contact (active infection or previous exposure) with disease agents in the Iberian lynx and sympatric carnivores at the study sites in Extremadura during 2014–2019 is expressed in Table 1. We consider active infection in the case of a positive PCR test, indicating the current presence of genetic material of the virus itself in the individual samples, and previous exposure in the case of a positive serological test, identifying the presence of antibodies against an infectious agent in the individual samples. In the case of real-time PCR, the cut-off for positivity was Ct < 35 (Ct is cycle threshold). We also included positive and negative controls for each run. In the conventional PCR, we also included positive and negative controls. In this case, the PCR product is observed as a band in the electrophoresis gel when the target is present in the sample. As for the ELISA tests, we considered positive results according to the manufacturer’s information. In the case of FHV-1, FCV and FPV, test kits are based on solid-phase immunoassay technology. The concentration of antibodies in serum samples is measured using the color-coded scale (“CombScale”) provided in the kit. The test kit results are documented in “S” units (ImmunoComb Score) on a scale of 0 to 6, where the positive value S3 corresponds to a 1:80 titer by virus neutralization test (VN) for FPV, 1:16 titer by VN for FHV-1 and 1:32 titer by VN for FCV. In the case of CDV, PV and SuHV-1 ELISA tests, we followed the manufacturer’s recommendations to differentiate seropositive and seronegative animals (positive threshold = sample optical density/positive control optical density >0.2, >0.15 and >0.35, respectively). For FeLV Agp27 and the FIV ELISA test, a positive test result was determined by color development in the sample spots from the device’s “Result Window”. Laboratory analyses were performed at the Centro de Analisis y Diagnostico (CAD, Malaga, Spain) and the Clinical Veterinary Hospital University of Extremadura (Caceres, Spain). These laboratories were selected because they are used by the Iberian lynx Ex-Situ Conservation Program, the Iberian Lynx Recovery Plan of Andalusia and the Iberian Lynx Reintroduction Program of Extremadura, accumulating two decades of experience in this felid.

For statistical analyses, lynxes were separated into three age classes: (1) juveniles living in the natal area (0–11 months), (2) subadults during the dispersal period (12–24 months) and (3) adults (>2 years old). They were also separated by sex; reintroduction/study site ((1) MRV, (2) ORV, (3) V or (4) VI); origin (captive-born versus wild-born) and year (2014–2019). The other species were divided just by the reintroduction/study area where they were sampled. In lynxes, prevalence differences between areas, sex, ages, origin and year were tested for using χ^2^ or Fisher’s exact test. For the rest of the carnivores, infectious disease prevalence differences between areas were tested using χ^2^ or Fisher’s exact test. For statistical analysis, animals that had at least one positive result were considered positive. Multiple samples from the same animal were not used to avoid pseudo-replication to ensure data independence [23]. Statistical analyses were performed with SAS 9.3 (SAS Institute, Cary, NC, USA).

Our research methodology was approved by the General Directorate of the Environment of Extremadura and according to the DOE 90 Iberian Lynx Recovery Plan in Extremadura (2016050104).

**Table 1:** Summary of methodologies used to detect the evidence of contact (active or previous) with disease agents in the Iberian lynx and sympatric carnivores.

Analyte	Number Tested ^a^	Sample	Assay	Detection	Name of Commercial Kits/Sequences (PCR)	Manufacturers and/or References
FHV-1	19 IL 40 FC	Serum	ELISA	Previous exposure	FHV Ab EIA F1007-AB02	Eurovet Veterinaria. [13]
					ImmunoCob Feline VacciChek Antibody Test Kit (FPV, FHV, FCV)	Biogal Galed Laboratories [24]
	67 IL	Orofaringeal Swab, clot, spleen	Real-time PCR	Active infection	FHV.351 F (5′-AGA GGC TAA CGG ACC ATC GA-3′) FHV.431 R (5′-GCC CGT GGT GGC TCT AAA-3′)	[25]
FCV	19 IL 40 FC	Serum	ELISA	Previous exposure	FCV Ab EIA F1008-AB02	Eurovet Veterinaria. [13] Biogal Galed Laboratories [24]
					ImmunoCob Feline VacciChek Antibody Test Kit (FPV, FHV, FCV)	
	67 IL	Orofaringeal Swab	Real-time RT- PCR	Active infection	FCV.F (5′-GTT GGA TGA ACT ACC CGC CAA TC-3′) FCV.R (5′-CAT ATG CGG CTC TGA TGG CTT GAA ACT G-3′)	[26]
PV	21 RF 11 SM 5 G 21 EM	Serum	ELISA	Previous exposure	Ingezim CPV	Ingenasa [27]
FPV	13 IL	Serum	ELISA	Previous exposure	ImmunoCob Feline VacciChek Antibody Test Kit (FPV, FHV, FCV)	Biogal Galed Laboratories [24]
	67 IL 40 FC	Rectal Swab, mesenteric ganglia	Real-time PCR	Active infection	PV3294 F (5′-ACT GCA TCA TTG ATG GTT GCA-3′) PV3400 R (5′-GGT ATG GTT GGT TTC CAT GGA-3′)	[28]
FCoV	52 IL	Serum	ELISA	Previous exposure	Ingezim FCoV© 16.FCV.K1	Ingenasa [13]
	67 IL	Rectal swab, clot, intestinal scrapping sample	Real-time RT- PCR	Active infection	FCoV1(1128) F (5′-AAC AAT CAC TAG ATC CAG ACG TTA GCT-3) FCoV2(1129) R (5′-GAT TTG ATT TGG CAA TGC TAG ATTT-3′)	[29]
CDV	52 IL 11 SM 40 FC 5 G 21 EM 21 RF	Serum	ELISA	Previous exposure	Ingezim Moquillo IgG^®^ 15.CDG.K1e	Ingenasa [13,14]
	67 IL 17 SM 75 FC 13 G 27 EM 26 D 1 EB 36 RF	Blood	Real-time RT- PCR	Active infection	CDV.78 F (5′ GGA AGC CTT GAT GAT AGC ACT GA 3′) CDV.161 R (5′-GCC GAA AGA ATA TCC CCA GTT-3′)	[19]
FeLV Ag p27	57 IL 60 FC	Serum	ELISA	Active infection	Snap Combo Plus	IDEXX Laboratories Inc.
FeLV provirus	67 IL 75 FC	Blood, clot, mesenteric ganglia, bone marrow	Real-time PCR	Active infection/regressive infection	exoFeLV-U3F2 (5′-AAC AGC AGA AGT TTC AAG GCC-3′) exoFeLV-U3R2 (5′-TTA TAG CAG AAA GCG CGC G-3′)	[30]
FIV	67 IL 40 FC	Serum	ELISA	Previous exposure	Snap Combo Plus	IDEXX Laboratories Inc.
	57 IL	Blood	Real-time PCR	Active infection	FIV.552f (5′-GCCTTCTCTGCAAATTTAACACCT-3′) FIV.672r (5′-GATCATATTCTGCTGTCAATTGCTTT3) ′FIV.582p (5′-6FAM-CATGGCCACATTAATAATGGCCGCA-TAMRA-3′)	[31]
*Leptospira* spp.	51 IL	Blood, Clot, renal tissue	Real-time PCR	Active infection	LipL32-45F (5′-AAG CAT TAC CGC TTG TGG TG-3′) LipL32-286R (5′-GAA CTC CCA TTT CAG CGA TT-3′)	[32]
*Cytauxzoon* spp.	15 IL	Blood	Conventional PCR	Active infection	Cytfelis.203 F (5′-AGA CCY YAA ACC ATC CCG CT-3′) Cytfelis.423 R (5′-CCT GCT GCC TTC CTT AGA TG-3′)	[33]
SuHV-1	17 IL	Cerebrum	Real-time PCR	Active infection	ADV-1F (5′ ATG GCC ATC TCG CGG TGC 3′) ADV-1R (5′ACT CGC GGT CCT CCA GCA 3′); ADV-2F (5′ACG GCA CGG GCG TGA TC 3′) ADV-2R (5′GG TTC AGG GTA CCC CGC 3′)	[34]
SuHV-1	17 IL	Serum	ELISA	Previous exposure	Ingezim ADV	Ingenasa

^a^ IL: Iberian lynx, FC: feral cat, SM: stone marten, D: dog, EM: Egyptian mongoose, EB: European badger and G: genet.

## 3. Results

In this study, the serological results should be interpreted with caution. Since there are no fully validated species-specific tests for this species, we tried to mimic the tests and methodology used in previous research with the Iberian lynx [13,14] or the closest relative, the sister taxa Eurasian lynx [24]. As there are no serological tests validated for the wild carnivores sampled in our study, we also used tests developed for domestic dogs and cats widely used in the testing of wild carnivores [14,35,36,37,38].

### 3.1. Active and Previous Infections in Iberian Lynxes

The prevalence of active infection in the Iberian lynx can be found in Table 2. The locations of positive lynxes are expressed in Figure 1b. Active infection of FPV, FeLV and *Cytauxzoon* sp. were each detected in three different lynxes of Matachel River Valley (MRV) (1.9%, 95% confidence interval = 0–5.7%, 1.9% 0–5.7 and 7.7% 0–22, respectively). SuHV-1 was also detected in the same male juvenile lynx coinfected with FeLV (7.1%, 0–20.6) [20]. Of the two lynxes, SuHV-1 was detected in the one from Valdecigueñas (V). The differences between areas were not statistically significant. All lynxes reported with active infections were dead at the time of the screening. From all of them, SuHV-1-infected lynxes died as a consequence of the disease. The FPV-infected lynx died because of a vehicle collision, and during necropsy, no signs associated to FPV were reported. The *Cytauxzoon* sp.-infected lynx died of a concurrent septicemia due to *Aeromonas veronii.*

Low rates of past contact with viral agents were identified in all the reintroduction/study sites (Table 2). Canine distemper virus (CDV; 5%, 0–11.8), feline calicivirus (FCV; 5.9%, 0–17.1), feline coronavirus (FCoV; 2.5%, 0–7.3) and FPV (8.3%, 0–24) were detected in lynxes from MRV. One lynx tested positive against FCV and FPV at this site. One out of three lynxes tested positive against CDV from the V study site. The differences between areas were not statistically significant. Significant age, sex or origin-related differences were not found for any of the positive-tested lynxes. Results of the statistical analysis are detailed in Table S1.

Since the duration of the antibody response against FHV-1, FCV and FPV after vaccination is unknown in the Iberian lynx, reintroduced individuals previously vaccinated against these agents (*n*= 40) were not included in the serological survey.

### 3.2. Active and Previous Infections in the Sympatric Carnivore Community

We detected the feline leukemia provirus in feral cats from three reintroduction sites (Table 2). The highest prevalence was found in ORV (22.7%, 5.2–40.2), followed by MRV (14.3%, 3.7–24.9) and V (9.1%, 0–26.1). From those sites, we only observed FeLV viremic cats in MRV (10.7%, 0–22.2). The differences between areas were not statistically significant.

The active infection of CDV was observed in a stone marten from V (20%, 0–55.1) and two red foxes from VI (20%, 0–44.8). The highest prevalence of CDV was found in VI (8%, 0–18.6), followed by V (3.2%, 0–9.4). There were significant differences in the CDV active infection prevalence between the reintroduction sites (χ^2^ =9.64, *p* = 0.02).

Antibodies to FHV-1, FCV and FPV were detected in feral cats from the two reintroduction sites where data was available. In MRV, the FHV-1 prevalence was 18.2%, 2.1–34.3, whereas, in ORV, it was 6.2%, 0–18.1. The FCV prevalence in MRV was 81.8%, 65.7–97.9 and, in ORV, was 31.2%, 8.5–54. In regard to FCV, the observed differences between the areas were statistically significant (χ^2^ = 9.91, *p* = 0.0016). The FPV prevalence in MRV was 12.5%, 0–25.7 and, in ORV, was 18.7%, 0–37.9. Moreover, the antibodies to CDV and the feline immunodeficiency virus (FIV) were observed in feral cats from MRV. Two cats tested positive against CDV (8.3%, 0–19.4), and one tested positive against FIV (4.2%, 0–12.2).

Antibodies to CDV were detected in four red foxes and one Egyptian mongoose from MRV and three common genets and eight red foxes from VI. Taking into consideration all the species, the highest CDV previous exposure prevalence corresponded to VI (44%, 24.5–63.5) versus MRV (13.5%, 4.2–22.7). The observed differences between the areas were statistically significant (χ^2^ = 16.5, *p* = 0.0003).

Antibodies to parvovirus were found in only one red fox (1/3) from ORV. Results of the statistical analysis are detailed in Table S2.

Thirteen privately owned dogs in which serum was available for the serological survey were not included in the study due to recent vaccinations against CDV and canine parvovirus (≤six months).

## 4. Discussion

Due to the conservation status, the small population size at the beginning of the program, and bearing in mind that each of the live captures performed must adhere to specific criteria according to the program’s demands (radio-collaring, health evaluation and emergency situation), our lynx sample was age-, sex- and site-biased.

This is the first report of a disease screening in a reintroduced population of Iberian lynxes. As it occurred in previous disease surveillances in the last strongholds of the species in Southern Spain (Doñana and Sierra Morena [13,14]), the prevalence of active and previous infections can be considered low in all reintroduction/study sites. Our results also concur with the surveys performed on the Eurasian lynx (*Lynx lynx* [24,39,40]), the Canada lynx (*L*. *canadensis* [41]) or the bobcat (*L. rufus* [42]). We believe that this may derive due to the solitary social structure of the species, which limits the frequency of intraspecific contacts ([13,43]). Another hypothesis that may explain the low seroprevalence includes the low rates of survival of lynxes infected with viruses [14] or that a reintroduced species may benefit from a temporary enemy release-like effect in the form of reduced parasite pressure, at least during a restricted period of time in which reintroduced native hosts are expected to encounter parasites that are well-adapted to exploiting them [11].

A remarkable difference from this study is that all reintroduced lynxes were vaccinated against the most common feline viral pathogens (FHV-1, FCV, FPV and FeLV), so conferring immunity from the vaccination may prevent the reintroduced lynxes from developing infections, at least during part of the time of the study. Once released, only a FeLV booster was administered to the reintroduced lynxes. Additionally, FeLV primo-vaccinations and boosters were administered to wild-born lynxes during the trapping season. This may explain the low contact rates with this agent in the reintroduced population at all reintroduction/study sites, even after contact with currently infected domestic cats during intraguild predations [44]. Only one juvenile wild-born lynx found dead was FeLV provirus-positive and coinfected with SuHV-1. FeLV was the only virus consistently detected in feral cats from all areas sampled. The prevalence ranged from 9–23%, which is higher compared to owned cats tested at the national level (2.6% (1.4–4.8) [45]), similar to what was found in the remnant lynx populations between 2004–2006 (23% [14]) and lower in domestic cats surveyed at the two Iberian lynx reintroduction areas from Andalusia (29.5% [46]). Since this virus was responsible for an outbreak in Doñana with devastating consequences, efforts to control this disease have been underway since the beginning of the reintroduction program by vaccinating lynxes, the control of feral cats and vaccinating farm-associated cats.

Serum from reintroduced and vaccinated lynxes was not included in the screening for FHV-1, FCV and FPV. Only serum available from lynxes born into the wild, which remained unvaccinated against these viruses, were assumed to be susceptible to the infection and could reflect the contact rate. Although this sample subset was small, we registered one previous exposure to FPV and FCV in one subadult lynx from MRV. Moreover, we also registered an active infection of FPV in a road-killed reintroduced subadult lynx. No lesions associated with this pathogen were observed during the necropsy, maybe because this finding occurred during an initial stage of the infection due to the protection generated by the previous vaccination or due to the ability to control the infection by the individual. The exposure to FPV in feral cats from this area reached 12.5% and FCV reached 82%. Unlike FCV, where direct contact is the typical mode of transmission, the feces of FPV-infected cats present long environmental survival, making indirect contact the most common and main transmission route [47]. We hypothesized that contact with this agent may be more frequent in areas where the prevalence in cats is high. Lower parvovirus (feline or canine, since the tests performed did not allow to differentiate the strain) contact rates were recorded in the sympatric wild carnivore community. The prevalence of the parvovirus in the carnivore community was lower compared to other serosurveys carried out in other lynx territories [14]. Since FPV has caused mortality in the Iberian lynx [15] and other lynx species (Eurasian lynx [39] and bobcat [48]), FPV routine monitoring should be continued in the lynxes and sympatric carnivores from the reintroduction/study sites. 

Although the lynx contact rates with FCV were considered low and similar to past research [13,14], FCV may be enzootic in the feral cat population from the sampled reintroduction sites, with the prevalence ranging from 31–82%. This prevalence is higher than those previously reported [14]. Most FCV strains induce a mild syndrome characterized by pyrexia, oral ulceration and mild respiratory and conjunctival signs; however, others are more virulent and may induce more severe systemic diseases, including high mortality (i.e., FCV-associated virulent systemic disease (FCV-VSD)) [49]. Monitoring FCV may be recommended during the first phases of the reintroduction to ensure that less virulent strains circulate among the lynxes and sympatric felids.

*Cytauxzoon* sp. DNA was found in one dead adult lynx. During necropsy, the lynx presented septicemia caused by the opportunistic bacterium *Aeromonas veronii.* Although cytauxzoonosis may be nonfatal in the Iberian lynx due to the lack of mortality evidence [15], we could not exclude the role of the *Cytauxzoon* sp. in the death of this lynx or the synergic role in this event of this particular coinfection. During the next live captures and necropsies, whenever possible, an attempt to screen for *Cytauxzoon* sp. was made, but the sample subset was small, and no evidence of current contact was registered. We also did not find high ticks loads in any of the individuals examined. Nevertheless, if ticks were found during live captures, fipronil (Frontline Spot-On Gato, Boehringer Ingelheim Vetmedica GmbH, Ingelheim Am Rhein, Germany) was administered topically. Previously, the *Cytauxzoon* sp. has only been recorded in Sierra Morena [27,50]. In order to infer a relevant understanding of the prevalence and pathogenicity of this piroplasm in the reintroduced population, screening may be continued. 

Apart from this opportunistic bacteria colonization, only one additional lynx suffered from a bacterial infection causing the death of the individual (*Streptococcus canis* necrotizing fasciitis/myositis [51]). In regard to bacteria, this study only included the screening of the current infection with *Leptospira* spp., yielding negative results. To gain more information about the impact in the population, a serological survey should be included in the future.

Previous exposure to CDV was recorded in MRV (5%; 2/40) and V (33%, 1/3), which differs from previous studies that failed to detect CDV antibodies [13,14]. In this case, our results are in consonance with those obtained by [50]. We may find higher CDV prevalence than in the former studies, since the reintroduction landscape was free from apex predators for decades, and under that scenario, meso-carnivores thrive, along with multi-host pathogens; higher lynx meso-predator contact rates may occur when the reintroduction begins [6]. During an ecological study in MRV, where changes in the carnivore community were examined before and after the Iberian lynx reintroduction, seventeen intraguild predations were detected [6,44]. These interactions well-provided the arena for the direct transmission of pathogens such as CDV. This virus was present in the carnivore community in three out of four reintroduction/study sites. At the VI study site, the CDV prevalence was significantly higher than the rest. Eighty-nine percent (8/9) of red foxes and 100% (3/3) of genets were positive. Two foxes were also CDV PCR-positive in this area. This differs from a study from two Iberian lynx reintroduction areas in Andalusia, where a current infection of CDV in a sample of 146 carnivores was not detected [46]. At MRV, 44% (4/9) of red foxes, 8% (2/24) of feral cats and 7% (1/14) of Egyptian mongooses were positive. The CDV prevalence was also higher in red foxes from lynx-occupied areas from previous studies [14]. Although serum samples from the V study site were not available, at this site, one stone marten was PCR-positive. In a previous study, [19] found high viral loads in a dead lynx, as well as a positive RT-PCR stone marten. Therefore, not only foxes but viverrids and mustelids may play a relevant role as CDV reservoirs in the reintroduction areas, and the impact of this disease in the reintroduced population should be maintained over time.

Antibodies against FCoV were detected in 2.5% (1/40) of lynxes tested in MRV. We could not detect antibodies or antigen-positive lynxes in the rest of the areas. This agrees with previous studies [13,14]. Our study could not explore the role of feral cats in regard to this virus.

The detection of SuHV-1 in a dead, wild-born nine-month-old Iberian lynx after almost two years of the reintroduction [20] was considered crucial in the disease surveillance program for the species in this region. Since the discovery, suitable samples from dead lynxes were subjected to screening of the pathogen, and, in 2019, a new case aroused. In this case, a reintroduced, adult female lynx succumbed to the disease. We performed a serological survey in a subset of samples that failed to detect antibodies. This is in agreement with the serological findings in another imperiled species, the Florida puma (*Puma concolor coryi*), where no evidence of prior exposure was observed in live-captured pumas and where Aujeszky’s disease may be a significant but underdiagnosed mortality factor in this species [52]. To date, from all wild felid species tested against this disease (Florida puma, Amur tiger (*Panthera tigris altaica*), far-eastern leopard (*Panthera pardus orientalis*), Eurasian lynx (*Lynx lynx*) and far-eastern wildcat (*Prionailurus bengalensis euptilurus*), just Amur tigers have shown antibodies to SuHV-1 [24]. Since wild boar are considered the main prey for this species [53], these results may be due to a natural coevolution of the predator with a common pathogen from its main prey or may be caused by the exposure of tigers to low pathogenic strains of SuHV-1, a mechanism described for nonfatal disease encounters in hunting dogs [54]. Although wild boar is not commonly included in the Iberian lynx diet, lynxes may occasionally consume carrion [55], and since indirect transmission may also occur through viral excretion by the host, without direct contact with the wild boars themselves [56], we deem that the sustained active surveillance of SuHV-1 is mostly desirable to get a better understanding of the epizootiology of Aujeszky’s disease in the Iberian lynx.

The impact of pathogens such as *Mycobacterium bovis* or *Sarcoptes scabiei*, which are proven to cause morbidity and/or mortality in this species [57,58], as well as others with unknown potential over the individual fitness or at the population level, such as SARS-CoV-2, is lacking in this study and should be considered in future research. 

## 5. Conclusions

Apparently, during the study years, infectious diseases did not pose a threat to the steady population growth of the Iberian lynx in this region. The low contact (current and previous) rates may be explained by structural complexities within the landscape [59], heterogeneity in individual host behaviors [60], variations in the infectious dose received, individual susceptibility and parasite strains or types [11]. Moreover, vaccination strategies implemented before the release of the individuals, as well as during the trapping season or handling under different scenarios, may be one of the outcomes of the low active infection rates to certain pathogens in this reintroduced population at the time of the study. Nevertheless, these low contact rates also suggest vulnerability and unpredictability to a disease outbreak and, along with the emergence of two cases of Aujeszky’s disease in this Iberian lynx landscape, highlight the vital role that a continuous disease surveillance plays in the Iberian lynx reintroduction program.

## Figures and Tables

**Table 2:** Prevalence of active infection or previous exposure to selected pathogens in the Iberian lynx and the sympatric carnivore community by reintroduction/study site.

Infectious Diseases								
	Prevalence of Active Infection (Positive/Examined (%))							
Agent/Location	Iberian Lynx	Domestic/Feral Cat	Domestic Dog	Red Fox	Common Genet	Stone Marten	Egyptian Mongoose	Eurasian Badger
Matachel River Valley								
FHV-1	0/52(0)	-	-	-	-	-	-	-
FCV	0/52(0)	-	-	-	-	-	-	-
FPV	1/52(1.9)	-	-	-	-	-	-	-
FCoV	0/52(0)	-	-	-	-	-	-	-
CDV	0/52(0)	0/42(0)	0/5(0)	0/19(0)	0/7(0)	0/5(0)	0/19(0)	0/1(0)
FIV	0/52(0)	-						
FeLV Ag p27	0/44(0)	3/28(10.7)	-	-	-	-	-	-
FeLV Provirus	1/52(1.9)	6/42(14.3)	-	-	-	-	-	-
*Leptospira* spp.	0/41(0)	-	-	-	-	-	-	-
*Cytauxzoon* sp.	1/13(7.7)	-	-	-	-	-	-	-
SuHV-1	1/14(7.1)	-	-	-	-	-	-	-
Ortiga River Valley								
FHV-1	0/4(0)	-	-	-	-	-	-	-
FCV	0/4(0)	-	-	-	-	-	-	-
FPV	0/4(0)	-	-	-	-	-	-	-
FCoV	0/4(0)	-	-	-	-	-	-	-
CDV	0/4(0)	0/22(0)	0/15(0)	0/3(0)	-	0/1(0)	-	-
FIV	0/4(0)	-						
FeLV Ag p27	0/4(0)	0/21(0)	-	-	-	-	-	-
FeLV Provirus	0/4(0)	5/22(22.7)	-	-	-	-	-	-
*Leptospira* spp.	0/1(0)	-	-	-	-	-	-	-
*Cytauxzoon* spp.	0/1(0)	-	-	-	-	-	-	-
SuHV-1	0/1(0)	-	-	-	-	-	-	-
Valdecigueñas								
FHV-1	0/5(0)	-	-	-	-	-	-	-
FCV	0/5(0)	-	-	-	-	-	-	-
FPV	0/5(0)	-	-	-	-	-	-	-
FCoV	0/5(0)	-	-	-	-	-	-	-
CDV	0/5(0)	0/11(0)	0/6(0)	0/4(0)	0/3(0)	1/5(20)	0/2(0)	-
FIV	0/5(0)							
FeLV Ag p27	0/3(0)	0/11(0)	-	-	-	-	-	-
FeLV Provirus	0/5(0)	1/11(9.1)	-	-	-	-	-	-
*Leptospira* spp.	0/4(0)	-	-	-	-	-	-	-
*Cytauxzoon* spp.	0/1(0)	-	-	-	-	-	-	-
SuHV-1	1/2(50)	-	-	-	-	-	-	-
Valdecañas/Ibores								
FHV-1	0/6(0)	-	-	-	-	-	-	-
FCV	0/6(0)	-	-	-	-	-	-	-
FPV	0/6(0)	-	-	-	-	-	-	-
FCoV	0/6(0)	-	-	-	-	-	-	-
CDV	0/6(0)	-	-	2/10(20)	0/3(0)	0/6(0)	0/6(0)	-
FIV	0/6(0)	-	-	-	-	-	-	-
FeLV Ag p27	0/6(0)	-	-	-	-	-	-	-
FeLV Provirus	0/6(0)	-	-	-	-	-	-	-
*Leptospira* spp.	0/5(0)	-	-	-	-	-	-	-
	**Prevalence of Previous Exposure (Positive/Examined (%))**							
**Agent/Location**	**Iberian Lynx**	**Domestic/FeralCat**	**Domestic Dog**	**Red Fox**	**Common Genet**	**Stone Marten**	**Egyptian Mongoose**	**Eurasian Badger**
Matachel River Valley								
FHV-1	0/17(0)	4/22(18.2)	-	-	-	-	-	-
FCV	1/17(5.9)	18/22(81.8)	-	-	-	-	-	-
PV/FPV	1/12(8.3)	3/24(12.5)	-	0/9(0)	0/2(0)	0/3(0)	0/14(0)	0/1(0)
FCoV	1/40(2.5)	-	-	-	-	-	-	-
CDV	2/40(5)	2/24(8.3)	-	4/9(44.4)	0/2(0)	0/3(0)	1/14(7.1)	0/1(0)
FIV	0/45(0)	1/24 (4.2)	-	-	-	-	-	-
SuHV-1	0/7(0)	-	-	-	-	-	-	-
Ortiga River Valley								
FHV-1	-	1/16 (6.2)	-	-	-	-	-	-
FCV	-	5/16 (31.2)	-	-	-	-	-	-
PV/FPV	-	3/16(18.7)	-	1/3(33.3)	-	0/2(0)	-	-
FCoV	0/3(0)	-	-	-	-	-	-	-
CDV	0/3(0)	0/16(0)	-	0/3(0)	-	0/2(0)	-	-
FIV	0/3(0)	0/16(0)	-	-	-	-	-	-
SuHV-1	0/2(0)	-	-	-	-	-	-	-
Valdecigueñas								
FHV-1	0/1(0)	-	-	-	-	-	-	-
FCV	0/1(0)	-	-	-	-	-	-	-
PV/FPV	-	-	-	-	-	-	-	-
FCoV	0/3(0)	-	-	-	-	-	-	-
CDV	1/3(33.3)	-	-	-	-	-	-	-
FIV	0/3(0)	-	-	-	-	-	-	-
SuHV-1	0/2(0)	-	-	-	-	-	-	-
Valdecañas/Ibores								
FHV-1	0/1(0)	-	-	-	-	-	-	-
FCV	0/1(0)	-	-	-	-	-	-	-
PV/FPV	0/1(0)	-	-	0/9(0)	0/3(0)	0/6(0)	0/7(0)	-
FCoV	0/6(0)	-	-	-	-	-	-	-
CDV	0/6(0)	-	-	8/9(88.9)	3/3(100)	0/6(0)	0/7(0)	-
FIV	0/6(0)	-	-	-	-	-	-	-
SuHV-1	0/6(0)	-	-	-	-	-	-	-

FHV-1: feline herpesvirus-1; FCV: feline calicivirus; PV: parvovirus; FPV: feline parvovirus; FCoV: feline coronavirus; CDV: canine distemper virus; FIV: feline immunodeficiency virus; FeLV Ag p27: feline leukemia virus antigen p27; SuHV-1: suid herpesvirus-1.

## Data Availability

Data supporting reported results are available upon request to authors.

## Supplementary Materials

The following are available online at https://www.mdpi.com/2076-2615/11/2/547/s1: Table S1: Statistical comparison (Fisher’s exact test) of the prevalence by age class, sex, study area, origin and sampling year in the Iberian lynx population. Table S2: Statistical comparison (Fisher’s exact test and chi-square test (1) of the prevalence by study area in the mesocarnivore community.
