# Supplementary material for: Disease Surveillance during the Reintroduction of the Iberian Lynx (Lynx pardinus) in Southwestern Spain
Source: Animals (Basel). 2021 Feb 19;11(2):547. doi: 10.3390/ani11020547 (PMC7923217; doi:10.3390/ani11020547)
Supplement: Supplementary file 1 [file animals-11-00547-s001.pdf]

animals

Supplementary Material

Disease Surveillance during the reintroduction of the Iberian Lynx (*Lynx pardinus*) in Southwestern Spain

Fernando Nájera [1,2,*], Rebeca Grande-Gómez [3,4], Jorge Peña [3], Anastasio Vázquez [3], María Jesús Palacios [5], Carmen Rueda [6], Ana Isabel Corona-Bravo [7], Irene Zorrilla [7], Luis Revuelta[1], María Gil-Molino [8] and José Jiménez [9]

[1] Department of Animal Physiology, Faculty of Veterinary Medicine, Complutense University of Madrid, 28040 Madrid, Spain; lrevuelt@vet.ucm.es
[2] Asistencia Técnica de la Dirección General del Medio Natural y Desarrollo Sostenible de la Junta de Comunidades de Castilla-La Mancha, Plaza del Cardenal Siliceo s/n, 45071 Toledo, Spain
[3] GPEX-Dirección General de Medio Ambiente, Junta de Extremadura, Avda. Luis Ramallo s/n, 06800 Mérida, Badajoz, Spain; rgrandegomez@gmail.com (R.G.-G); jorgepmartinez@gmail.com (J.P.); tasiopeseto@gmail.com (A.V.)
[4] Organismo Autónomo Parques Nacionales, Zarza de Granadilla, 10710 Cáceres, Spain
[5] Dirección General de Medio Ambiente de la Junta de Extremadura, Avda. Luis Ramallo, s/n, 06800, Mérida, Badajoz, Spain; mariajesus.palacios@juntaex.es
[6] Fundación CBD-Hábitat, C/Gustavo Fernández Balbuena 2, Entreplanta, Oficina A, 28002 Madrid, Spain; carmen.rueda91@gmail.com
[7] Centro de Análisis y Diagnóstico de la Fauna Silvestre, Agencia de Medio Ambiente y Agua de Andalucía, Consejería de Agricultura, Ganadería, Pesca y Desarrollo Sostenible, Junta de Andalucía, Avenida Lope de Vega 9, 29010 Málaga, Spain; aisabel.corona@juntadeandalucia.es (A.I.C.-B.); irene.zorrilla.delgado@juntadeandalucia.es (I.Z.)
[8] Servicio de Recepción y Diagnostico de Muestras Biológicas, Hospital Clínico Veterinario, Universidad de Extremadura, Avda. Universidad s/n, 10003 Cáceres, Spain; magilmo84@gmail.com
[9] Instituto de Investigación en Recursos Cinegéticos-(CSIC-UCLM-JCCM), 13071 Ciudad Real, Spain; Jose.Jimenez@uclm.es
* Correspondence: fernanaj@ucm.es

Citation: Nájera, F.; Grande-Gómez, R.; Peña, J.; Vázquez, A.; Palacios, M.J.; Rueda, C.; Corona-Bravo, A.I.; Zorrilla, I.; Revuelta, L.; Gil-Molino, M. et al. Disease surveillance during the reintroduction of the Iberian lynx (*Lynx pardinus*) in Southwestern Spain. *Animals* **2021**, *11*, 547. https://doi.org/10.3390/ani11020547

Academic Editor: Luigi Boitani

Received: 12 January 2021
Accepted: 12 February 2021
Published: 19 February 2021

Publisher's Note: MDPI stays neutral with regard to jurisdictional claims in published maps and institutional affiliations.

Copyright: © 2021 by the authors. Licensee MDPI, Basel, Switzerland. This article is an open access article distributed under the terms and conditions of the Creative Commons Attribution (CC BY) license (http://creativecommons.org/licenses/by/4.0/).

Table S1. Statistical comparison (Fisher's Exact Test) of prevalence by age class, sex, study area, origin, and sampling year in the Iberian lynx population.

Statistical comparison	Results
**FeLV: Feline Leukemia Virus prevalence (Active infection)**	
Lynx-Age (juvenile, subadult, adult)	N.S. (p = 0.5224)
Lynx-Sex (male, female)	N.S. (p = 1.0000)
Lynx-Area (Matachel, Ortiga, Valdecigüeñas, Valdecañas)	N.S. (p = 1.0000)
Lynx-Origin (captive vs wild-born)	N.S. (p = 0.4179)
Lynx-Sampling year (2015,2016,2017,2018, 2019)	N.S. (p = 0.3731)
**FPV: Feline Parvovirus prevalence (Active infection)**	
Lynx-Sampling year (2015,2016,2017,2018, 2019)	N.S. (p = 0.3731)
Lynx-Sex (male, female)	N.S. (p = 1.0000)
Lynx-Age (juvenile, subadult, adult)	N.S. (p = 0.5224)
Lynx-Area (Matachel, Ortiga, Valdecigüeñas, Valdecañas)	N.S. (p = 1.0000)
Lynx-Origin (captive vs wild-born)	N.S. (p = 1.0000)
**SuHV-1: Suid Herpesvirus 1 prevalence (Active infection)**	
Lynx-Sampling year (2015,2016,2017,2018, 2019)	N.S. (p = 0.4853)
Lynx-Sex (male, female)	N.S. (p = 1.0000)
Lynx-Age (juvenile, subadult, adult)	N.S. (p = 0.4853)
Lynx-Area (Matachel, Ortiga, Valdecigüeñas, Valdecañas)	N.S. (p = 0.3309)
Lynx-Origin (captive vs wild-born)	N.S. (p = 1.0000)
***Cytauxzoon sp.* prevalence (Active infection)**	
Lynx-Sampling year (2015,2016,2017,2018, 2019)	N.S. (p = 0.1333)
Lynx-Sex (male, female)	N.S. (p = 0.4667)
Lynx-Age (juvenile, subadult, adult)	N.S. (p = 0.5333)
Lynx-Area (Matachel, Ortiga, Valdecigüeñas, Valdecañas)	N.S. (p = 1.0000)
Lynx-Origin (captive vs wild-born)	N.S. (p = 1.0000)
**FCV: Feline calicivirus prevalence (Previous exposure)**	
Lynx-Sampling year (2015,2016,2017,2018, 2019)	N.S. (p = 0.0526)
Lynx-Sex (male, female)	N.S. (p = 1.0000)
Lynx-Age (juvenile, subadult, adult)	N.S. (p = 1.0000)
Lynx-Area (Matachel, Ortiga, Valdecigüeñas, Valdecañas)	N.S. (p = 1.0000)
**FPV: Feline Parvovirus prevalence (Previous exposure)**	
Lynx-Sampling year (2015,2016,2017,2018, 2019)	N.S. (p = 0.0769)
Lynx-Sex (male, female)	N.S. (p = 1.0000)
Lynx-Age (juvenile, subadult, adult)	N.S. (p = 1.0000)
Lynx-Area (Matachel, Ortiga, Valdecigüeñas, Valdecañas)	N.S. (p = 1.0000)
**FCoV: Feline Coronavirus prevalence (Previous exposure)**	
Lynx-Sampling year (2015,2016,2017,2018, 2019)	N.S. (p = 0.0769)
Lynx-Sex (male, female)	N.S. (p = 0.4231)
Lynx-Age (juvenile, subadult, adult)	N.S. (p = 0.5000)
Lynx-Area (Matachel, Ortiga, Valdecigüeñas, Valdecañas)	N.S. (p = 1.0000)
Lynx-Origin (captive vs wild-born)	N.S. (p = 0.4038)
**CDV: Canine Distemper Virus prevalence (Previous exposure)**	
Lynx-Sampling year (2015,2016,2017,2018, 2019)	N.S. (p = 1.0000)
Lynx-Sex (male, female)	N.S. (p = 0.5670)
Lynx-Age (juvenile, subadult, adult)	N.S. (p = 0.1824)
Lynx-Area (Matachel, Ortiga, Valdecigüeñas, Valdecañas)	N.S. (p = 0.2468)
Lynx-Origin (captive vs wild-born)	N.S. (p = 1.0000)

Table S2. Statistical comparison (Fisher's Exact Test and Chi-squared test (1) of prevalence by study area in the mesocarnivore community.

Statistical comparison	Test result
**CDV: Canine Distemper Virus prevalence (Active infection)**	
Mesocarnivore-Area (Matachel, Ortiga, Valdecigüeñas, Valdecañas) (1)	$\chi^2 = 9.64$, $p = 0.02$
**CDV: Canine Distemper Virus prevalence (Previous exposure)**	
Mesocarnivore-Area (Matachel, Ortiga, Valdecigüeñas, Valdecañas) (1)	$\chi^2 = 16.5$, $p = 0.0003$
**FeLV: Feline Leukemia Virus prevalence (Active infection)**	
Mesocarnivore-Area (Matachel, Ortiga, Valdecigüeñas, Valdecañas)	N.S. ($p = 0.6721$)
**FeLV Agp27: Feline Leukemia Virus Antigen p27 prevalence (Active infection)**	
Mesocarnivore-Area (Matachel, Ortiga, Valdecigüeñas, Valdecañas)	N.S. ($p = 0.2857$)
**PV: Parvovirus prevalence (Previous exposure)**	
Mesocarnivore-Area (Matachel, Ortiga, Valdecigüeñas, Valdecañas)	N.S. ($p = 0.0862$)
**FPV: Feline Parvovirus prevalence (Previous exposure)**	
Mesocarnivore-Area (Matachel, Ortiga, Valdecigüeñas, Valdecañas)	N.S. ($p = 0.6678$)
**FHV-1: Feline Herpesvirus 1 prevalence (Previous exposure)**	
Mesocarnivore-Area (Matachel, Ortiga, Valdecigüeñas, Valdecañas)	N.S. ($p = 0.3741$)
**FCV: Feline Calicivirus prevalence (Previous exposure)**	
Mesocarnivore-Area (Matachel, Ortiga, Valdecigüeñas, Valdecañas)(1)	$\chi^2 = 9.91$, $p = 0.0016$
**FIV: Feline Immunodeficiency Virus prevalence (Previous exposure)**	
Mesocarnivore-Area (Matachel, Ortiga, Valdecigüeñas, Valdecañas)	N.S. ($p = 1.0000$)

N.S.: No significant.
